# Ultrasound-guided Erector Spinae Plane Block in a Child Undergoing Laparoscopic Cholecystectomy

**DOI:** 10.7759/cureus.2241

**Published:** 2018-02-27

**Authors:** David Terence Thomas, Serkan Tulgar

**Affiliations:** 1 Department of Pediatric Surgery, Maltepe University Faculty of Medicine; 2 Department of Anesthesiology & Reanimation, Maltepe University Faculty of Medicine

**Keywords:** mutlimodal analgesia, erector spinae plane block, pediatric laparoscopy, cholecystectomy

## Abstract

Erector spinae plane block (ESP) is a recently described regional anesthesia technique that leads to the blockage of both visceral and somatic nerve fibers. While there are anecdotal reports of ESP used in children, none are for laparoscopic procedures. Herein we report a child undergoing laparoscopic cholecystectomy in which ESP was used as part of multimodal anesthesia. Ultrasound-guided ESP block is an easily performed peripheral nerve block that leads to long-lasting postoperative analgesia. It can be successfully used in pediatric laparoscopic procedures such as cholecystectomy and should be kept in mind as an option for multimodal analgesia in children.

## Introduction

Erector spinae plane block (ESP) is a recently described regional anesthesia technique [1]. ESP has been reported to be suitable for laparoscopic upper abdominal surgery as it blocks both somatic and visceral pain [2]. Laparoscopic cholecystectomy is a common surgical procedure in adults and is less frequently performed in children. While there are anecdotal reports of ESP used in children, none are for laparoscopic procedures [3,4]. Herein, we would like to report our experience of ESP block used for postoperative analgesia in a child undergoing laparoscopic cholecystectomy for cholelythiasis. Written informed consent was obtained from the child’s legal guardian for all procedures and for use of case data in this report.

## Case presentation

An 11-year-old American Society of Anesthesiology (ASA) class 1 patient (23 kg, 140 cm) was scheduled to undergo laparoscopic cholecystectomy due to cholelythiasis and recurrent attacks of cholecystitis. The patient’s medical history revealed previous tonsillectomy at the age of 6 years, during which time an allergic reaction to opioids occurred. Therefore, ESP block was chosen for perioperative analgesia management, in addition to 10 mg/kg of intravenous paracetamol. Following opioid-free anesthesia induction and intubation, the patient was placed in the lateral position following hemodynamic stability. ESP block was performed using sterile techniques. Following skin prep using 10% povidone-iodine, a high-frequency linear ultrasound transducer was placed over the spinous process of the 9th thoracic vertebra in the sagittal plane. The transducer was moved 2.5 cm laterally on the parasagittal plane to visualise to transverse process. A total of 15 ml of 0.25% bupivacaine was administered between the transverse process and erector spinae muscles using an insulated needle. The same procedure was performed for the opposite side. The patient was then placed in the supine position. Laparoscopic cholecystectomy was performed using the standard five trocar technique, using 10 mm H2O insufflation pressure. Surgical procedure lasted 107 minutes. Following extubation, the patient was transferred to the recovery room where the patient had no nausea and his Numeric Rating Scale (NRS) was 0/10. The patient was transferred to the ward. Hourly NRS follow-up revealed NRS < 3 during the first 24 hours. Scheduled paracetamol 3 × 10 mg/kg was not administered. The patient was discharged on the postoperative second day with oral paracetamol for analgesia.

## Discussion

Our case has demonstrated that ultrasound-guided ESP block can be successfully used for long-lasting analgesia in pediatric laparoscopic cholecystectomy. Such laparoscopic procedures lead to somatic pain from skin incisions and visceral pain from peritoneal irritation due to carbon dioxide insufflation.

In laparoscopic cholecystectomy, multimodal analgesia generally consists of a combination of paracetamol, non-steroidal anti-inflammatory drugs, and opioids. Transversus abdominis plane block, paravertebral block, and oblique subcostal transversus abdominis plane block may also be used [5,6]. However, apart from paravertebral block, none of these regional anesthesia techniques lead to visceral pain block. ESP blocks both visceral and somatic nerve fibers and mechanical complications are rare as local anesthesia application is performed distant to any nerve or vital organ. Our report has demonstrated that ultrasound-guided ESP block is a safe and effective technique. For these reasons, ESP block should be considered as a regional anesthesia component of multimodal analgesia in pediatric laparoscopic procedures such as cholecystectomy. However, further controlled, randomized and blinded studies are required to provide further evidence of ESP block’s effectiveness and safety that may lead to its routine use in children.

## Conclusions

Ultrasound-guided ESP block is an easily performed peripheral nerve block that leads to long-lasting postoperative analgesia. It can be successfully used in pediatric laparoscopic procedures such as cholecystectomy and should be kept in mind as an option for multimodal analgesia in children.
